# First Report on Wild Ginger (Family: Zingiberaceae) Species Composition with New Records in Limestone Forests of Kelantan, Peninsular Malaysia

**DOI:** 10.21315/tlsr2022.33.3.3

**Published:** 2022-09-30

**Authors:** Suganthi Appalasamy, Nivaarani Arumugam, Nor Syahaiza Ahmad Zamri, Anis Fadhlina, Jayaraj Vijaya Kumaran, Sreeramanan Subramaniam

**Affiliations:** 1 Institute of Food Security and Sustainable Agriculture, Universiti Malaysia Kelantan, Jeli Campus, 17600 Jeli, Kelantan, Malaysia; 2 Faculty of Earth Science, Universiti Malaysia Kelantan, Jeli Campus, 17600 Jeli, Kelantan, Malaysia; 3 School of Biological Sciences, Universiti Sains Malaysia, 11800 USM Pulau Pinang, Malaysia; 4 Chemical Centre Biology, Universiti Sains Malaysia, 11900 Bayan Lepas, Pulau Pinang, Malaysia

**Keywords:** Distribution, Limestone, Malaysia, Plant, Wild Ginger, Zingiberaceae, Taburan, Batu Kapur, Malaysia, Tumbuhan, Halia Hutan, Zingiberaceae

## Abstract

The wild gingers in the family Zingiberaceae have a wide range of habitat distribution. The species growing in Malaysian forests are the most studied. Nevertheless, the aromatic perennial herb family found in limestone forests is the least studied. The present study identified the ginger species compositions, determined the conservation status of the identified ginger species, and compared the distribution of the ginger species in selected limestone forests of Kelantan due to the lack of intensive study focusing on wild gingers in Malaysian limestone forests, especially in the state of Kelantan, to date. In various months, wild ginger species observation was conducted at four limestone forests in Kelantan. From the survey performed during the present study, Gua Setir and Gua Ikan recorded 16 species with 12.5% overlapping species. Gua Setir comprised 61.5% more ginger species than Gua Ikan. In total, 13 species (81.25%) were evaluated based on the Red List of Threatened Species by the International Union for Conservation of Nature (IUCN). Three wild ginger species listed as high conservation value (HCV), *Zingiber aurantiacum*, *Zingiber petiolatum* and *Zingiber wrayi*, were identified at the limestone karst valley of Gua Setir. The current study presented updated and new records of the limestone wild ginger flora in Kelantan. The research also demonstrated that each limestone forest consisted of different combinations of ginger species. Consequently, conservation efforts and sustainable management currently enforced in the limestone forests would lead to long-term protection of the plants. Furthermore, the wild gingers could become a tourist attraction for limestone forests located in recreational areas.

## Abstract (fr)

Halia hutan (Famili: Zingiberaceae) mempunyai taburan habitat yang luas. Kajian mengenai tumbuhan herba yang berbau harum ini kurang dilakukan di kawasan hutan batu kapur Malaysia. Kajian ini telah mengenal pasti komposisi spesies halia, menentukan status pemuliharaan spesies halia yang telah dikenal pasti, serta membandingkan taburan spesies halia di beberapa hutan batu kapur di Kelantan. Kajian ini juga dilakukan kerana kekurangan kajian intensif yang memfokuskan halia hutan di hutan batu kapur Malaysia, terutamanya di negeri Kelantan, sehingga kini. Pemerhatian terhadap spesies halia hutan telah dilakukan di empat hutan batu kapur di Kelantan. Gua Ikan dan Gua Setir telah merekodkan 16 spesies halia semasa pemerhatian tersebut dengan 12.5% spesies yang bertindih. Gua Setir merangkumi 61.5% spesies halia berbanding Gua Ikan. Secara keseluruhan, 13 spesies (81.25%) halia yang dikenal pasti telah dinilai berdasarkan *Red List of Threatened Species* oleh International Union for Conservation of Nature (IUCN). Sebanyak tiga spesies halia telah disenaraikan sebagai spesies *high conservation value* (HCV) iaitu *Zingiber aurantiacum, Zingiber petiolatum* dan *Zingiber wrayi*. Ketiga-tiga spesies ini telah dikenal pasti di lembah kars Gua Setir. Kajian ini telah memberikan rekod spesies yang telah dikemas kini serta rekod baru bagi spesies halia hutan di kawasan batu kapur di Kelantan. Kajian ini juga menunjukkan bahawa setiap hutan batu kapur terdiri daripada gabungan spesies halia yang berbeza. Oleh yang demikian, usaha pemuliharaan dan pengurusan mampan yang dilakukan pada masa kini di hutan batu kapur akan membawa kepada perlindungan jangka panjang untuk tumbuhan halia. Tambahan pula, tumbuhan halia hutan juga boleh dijadikan sebagai tarikan pelancong untuk hutan batu kapur di kawasan rekreasi.

**Highlights**
- A total of 16 ginger species were recorded from limestone forest in Kelantan (Gua Ikan dan Gua Setir) with 15 new records.
- Three wild ginger species from Gua Setir are listed as high conservation value (HCV) species, namely *Zingiber aurantiacum*, *Zingiber petiolatum* and *Zingiber wrayi*.
- Each limestone forest consisted of different combinations of ginger species.

## INTRODUCTION

The gingers from the family Zingiberaceae are known as a herb worldwide. The perennial plant is generally distributed from lowland to hill forests (Larsen *et al*. 1999). Approximately 1,600 species of ginger are recorded worldwide (Larsen *et al*. 1999; Xu & Chang 2017), with over 160 species from 18 genera were documented in Peninsular Malaysia alone. Various studies were conducted around Malaysia to update the species composition and distribution of the Malaysian ginger plants. Among the investigations, Appalasamy *et al*. (2019), Appalasamy and Arumugam (2020), Appalasamy, Arumugam, et al. (2020), Appalasamy, Rathamanalan, et al. (2020) and Izlamira *et al*. (2020) recently published ginger species composition data for a specific area in Malaysia. Nonetheless, numerous forested areas in Malaysia are unexplored.

According to Larsen *et al*. (1999), gingers have a wide range of habitats, including limestone areas. Nevertheless, the research on limestone gingers is scarce, especially in Peninsular Malaysia. Chin (1977) reported 16 wild gingers species in the limestone areas in Peninsular Malaysia. Moreover, Kiew *et al*. documented two rare limestone ginger species in Peninsular Malaysia in 2017. As the total area of limestone hills in Malaysia has reduced due to anthropogenic activities (Liew *et al*. 2016), it is imperative to document the ginger species composition for future reference and support conservation activities.

Kelantan is reported to possess more limestone outcrops than any other state in Peninsular Malaysia, including Gua Madu, Gua Setir and Gua Ikan (Liew *et al*. 2021a; 2021b). The updated floral data on Kelantan limestone outcrops was published by Davison and Kiew (1990), which reported 210 flora in 120 limestone hills. To date, Chin (1983), Davison and Kiew (1990), and Kiew *et al*. (2017; 2019) are the only published articles that documented Kelantan limestone gingers. Accordingly, the present study aims to identify ginger species composition, determine the conservation status of the identified ginger species, and compare the distribution of ginger species in selected Kelantan limestone forests. The current study is essential to update the list of ginger species in specific habitats and contribute to conservation status analysis of a species at the global and local levels.

## MATERIALS AND METHODS

### Study Sites

The current study was conducted in four limestone forests between 2020 and 2021 as shown in Table 1. The observation periods were varied between the sites. The different observation time was due to environmental factors, such as flood and travel restrictions ascribable to the COVID-19 pandemic.

Continuous accessibility was the criterion for limestone forest selection. Consequently, the study sites were outside protected forests and accessible to everyday people. The global positioning system (GARMIN GPSMAP 64s, Malaysia) was employed to tag the location and elevation of the study sites.

## DATA COLLECTION

The present study surveyed the limestone karst valleys and forests within a 100 m radius of the karsts, and a random sampling method was performed. Ginger plants along nature trails, rivers, streams and limestone karst were observed and recorded in the study sites. The identification guides by Larsen *et al*. (1999), Theilade (1996; 1999), Khaw (2001), Kress *et al*. (2002), Poulsen (2006), Lamb *et al*. (2013) and de Boer *et al*. (2018) were referred. Species identification was conducted based on morphological characteristics, such as leaves, inflorescence and flowers.

A Canon (Japan) digital camera with a Raynox portable super macro conversion lens (Japan) was employed to photograph the identified plant species in the field. Plant specimens were collected for species that encompassed more than three colonies. The herbaria specimens were dried and deposited at the Natural Resources Museum, Faculty of Earth Science, Universiti Malaysia Kelantan.

### Data Analysis

The conservation status of the identified ginger species was analysed according to the Red List of Threatened Species by the International Union for Conservation of Nature (IUCN, 2021) as the global standard and Malaysia Red List (Yong *et al*. 2021) for local standards. Moreover, the species listed as High Conservation Value (HCV) was evaluated based on the Rare, Threatened and Endangered (RTE) species reported by the HCV Malaysia Toolkit Steering Committee (2018). The analyses were performed for only 15 ginger plants identified within the species level.

## RESULTS

**Figure 1:**
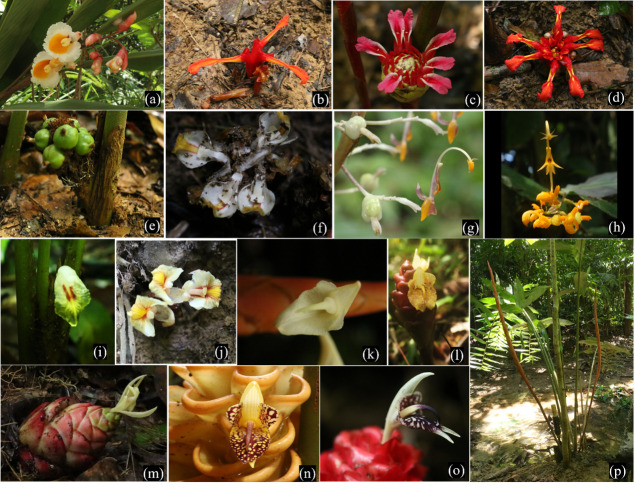
Identified wild ginger species in limestone forest of Kelantan. (a) *Alpinia javanica*; (b) *Etlingera littoralis*; (c) *Etlingera maingayi*; (d) *Etlingera punicea*; (e) *Plagiostachys* sp.; (f) *Wurfbainia uliginosa*; (g) *Globba leucantha*; (h) *Globba patens*; (i) *Bosenbergia plicata*; (j) *Sundamomum hastilabium*; (k) *Zingiber aurantiacum*; (l) *Zingiber ottensii*; (m) *Zingiber puberulum*; (n) *Zingiber spectabile*; (o) *Zingiber wrayi*; (p) *Zingiber petiolatum*.

A total of 16 ginger species from eight genera were identified from four limestone forests around Kelantan (see Fig. 1). Taxonomic identification was performed at the species level for 15 ginger plants, while one ginger plant was identified as a morphospecies. According to Kress *et al*. (2002) and de Boer *et al*. (2018), the species composition comprised three tribes, Alpinieae, Globbeae and Zingiberaceae (see Table 2).

**Figure 2:**
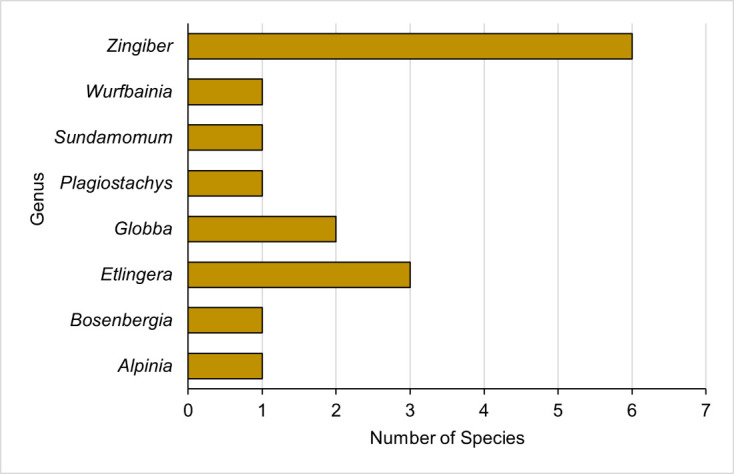
Species composition of gingers according to genus at limestone forests of Kelantan, Peninsular Malaysia.

The Zingiberaceae and Alpinieae gingers recorded seven species, respectively, from two and five genera. Meanwhile, the Globbeae tribe recorded two species. Among the tribes, Alpinieae recorded the highest number of genera. The genus *Zingiber* dominated the species composition with six species (see Fig. 2). The present study also recorded three *Etlingera* and two *Globba* species. Moreover, *Alpinia*, *Bosenbergia*, *Plagiostachys, Sundamomum* and *Wurfbainia* comprised single species.

The positions of the inflorescence in the gingers studied were categorised into five groups. Based on the classification of the inflorescence positions described by Holtum (1950) and Larsen *et al*. (1999), the gingers discovered in the current study were grouped as follows:

1. A terminal on leafy shoots: The ginger species under this category had inflorescence occurring on the terminal leafy shoots. A long peduncle with bracts was observed on the distal parts. The species in this group were *Alpinia javanica*, *Globba leucantha* and *Globba patens*.
2. A terminal on erect stems: The inflorescence of the observed ginger species broke through the sides of the sheaths of leafy stems, appearing on the lateral parts of leafy shoots. The species grouped under this category was *Plagiostachys* sp.
3. Radical (ground): The inflorescence was spotted on the ground, visible either near or to some extent from leafy shoots. The species in this classification were *Etlingera littoralis*, *E. punicea*, *Sundamomum hastilabium*, *Wurfbainia uliginosa*, *Zingiber aurantiacum*, *Z. puberulum* and *Z. wrayi*.
4. Radical (borne on a peduncle): The ginger species in this category exhibited a peduncle supported inflorescence. The length of the peduncles varied between species. The species that belonged in this group were *E. maingayi*, *Z. ottensii*, *Z. petiolatum* and *Z. spectabile*.
5. Between the leaves: The flowers (or, mainly, a single flower at a time) of *Bosenbergia plicata* was observed emerging between leaves and enclosed by leaf sheaths.

The current study conducted a conservation status analysis on 15 species identified at the species level as shown in Table 2. Among the species, 13 (81.25%) were listed in the Red List of Threatened Species reported by the IUCN, the global standard. Conversely, none of the studied species was registered in the Malaysia Red List. The highest number of species was categorised as Least Concern (LC) (seven), followed by two species under Data Deficient (DD) and Vulnerable (VU), and one species under Near Threatened (NT) and Endangered (EN). Furthermore, three wild ginger species, *Z. aurantiacum*, *Z. petiolatum* and *Z. wrayi*, were identified as High Conservation Value (HCV).

Table 3 summarises the ginger species distribution in Kelantan limestone forests. Gua Setir recorded a higher number of wild ginger species at 13 than five documented in Gua Ikan. Gua Setir also exhibited more genera than Gua Ikan. Seven genera were recorded in Gua Setir, namely *Alpinia*, *Bosenbergia*, *Etlingera*, *Globba*, *Plagiostachys*, *Wurfbainia* and *Zingiber*. Four genera, *Etlingera*, *Sundamomum*, *Wurfbainia* and *Zingiber*, were documented in Gua Ikan. Gua Setir was dominated by *Zingiber* with five species, while Gua Ikan comprised primarily of *Etlingera* with three species. A few ginger plants were also observed in Gua Madu and Gunung Reng. Nevertheless, the taxa of the gingers discovered in both sites were not identified as the ginger plants were infertile.

## DISCUSSION

The recorded 16 ginger species from eight genera in the limestone forests of Kelantan represented approximately 10% of the species and 44.44% of the ginger genera reported in Peninsular Malaysia (see Fig. 2). The gingers in genera *Etlingera*, *Globba* and *Zingiber* were previously documented in Kelantan limestone forests (Davison & Kiew 1990; Kiew *et al*. 2019). Two genera were identified as morphospecies, while one genus was identified at the species level.

The *Globba* and *Zingiber* morphospecies were reported in the Federal Land Development Authority (FELDA) Chiku and Relai Forest Reserve limestone hills, respectively (Kiew *et al*. 2019). According to Davison and Kiew (1990), *Etlingera maingayi* was distributed in the limestone hills of Gua Renayang and Pulau Raba, Kelantan. An endemic species to Peninsular Malaysia, *Boesenbergia longipes*, and a rare species, *Globba albiflora*, were also previously observed in the limestone areas in Kelantan (Kiew *et al*. 2017; 2019). Nevertheless, none of the species was recorded during the present study. Nonetheless, the current study updates the Zingiberaceae species list of the Kelantan limestone forests with 15 new records (see Table 3). Future ginger species studies with a more extended observation period in the limestone areas could reveal more new records.

In the current study, a morphospecies from the genus *Plagiostachys* was identified. Nevertheless, the absence of flowers in *Plagiostachys* sp. during sampling limited species identification as the structure of the inflorescence and flower are crucial morphological characteristics of ginger plants to confirm plant identification at the species level (Larsen *et al*. 1999). Interestingly, *Plagiostachys* was identified with a unique inflorescence structure that appeared to penetrate from the side sheaths of its leafy stems (Smith 1990; Larsen *et al*. 1999), as shown in Fig. 1e. Additionally, infructescence with green fruits was observed, requiring further identification. Recently, new wild ginger species from genera *Scaphochlamys* (Sam & Saw 2005; Sam *et al*. 2015) and *Globba* (Sam & Ibrahim 2016) were discovered in eastern Peninsular Malaysia. Thereby, *Plagiostachys* sp. from the current study could be added to the taxon of wild ginger species in Peninsular Malaysia as only three species of *Plagiostachys* (*P. albiflora*, *P. lateralis* and *P. mucida*) was previously observed (Larsen *et al*. 1999; Newman *et al*. 2004).

A higher number of Alpinieae and Zingiberaceae tribes than Globbeae were recorded in the study sites in the present study. Furthermore, Alpinieae dominated the species composition with more genera (62.5%), as shown in Table 3. Similar observations were also documented in other Kelantan forests. For example, the Alpinieae tribe comprised 71.4% of the genera from the overall species composition at the nature trail in Lojing Highlands (Appalasamy, Rathamanalan, et al. 2020). The tribe also comprised 50% of the wild ginger genera recorded in the Ulu Sat Forest Reserve (Izlamira *et al*. 2020).

The present study recorded more species in the genus *Zingiber* from the Zingiberaceae tribe (see Fig. 2). The genus is commonly found in limestone and lowland forests of Peninsular Malaysia. The highest number of *Zingiber* spp. (five species) was recorded in Gua Setir compared to other sites in Peninsular Malaysia, such as Lojing Highlands (two species) (Appalasamy, Rathamanalan, et al. 2020), Mount Telapak Buruk (one species) (Appalasamy & Arumugam 2020), and Pangkor Island (one species) (Appalasamy *et al*. 2019).

Chin (1983) stated that *Globba patens* and *Zingiber spectabile* were distributed in Peninsular Malaysia and recorded in limestone areas. Meanwhile, *Alpinia javanica*, *Wurfbainia uliginosa* and *Etlingera punicea* species were widespread in Peninsular Malaysia. Nonetheless, the distribution status of the other species recorded in Table 2 is still being updated (Larsen *et al*. 1999; Yong *et al*. 2021).

Three species were categorised as High Conservation Value (HCV) in the current study based on the IUCN (2021) and HCV Malaysia Toolkit Steering Committee (2018). The species were *Z. aurantiacum*, *Z. petiolatum* and *Z. wrayi*. The HCV species were not documented in other Kelantan limestone and lowland forests to date (Henderson 1939; Kiew *et al*. 2017; Izlamira *et al*. 2020). The absence of the HCV species in previous records might be due to the lack of sampling and low population size.

The wild gingers in several Kelantan forests remain undiscovered. According to Aliaa-Athirah *et al*. (2019), the lack of sampling led to low floral diversity, especially in Kelantan limestone hills. Furthermore, a genus revision based on the morphological characteristics of *Zingiber* by Theilade in 1996 proved that the HCV species were distributed in Peninsular Malaysia. Consequently, further wild ginger ecological studies in other parts of Kelantan could update the distribution status of the HCV species.

The IUCN conservation status demonstrated that the HCV species were present in low abundance globally. As a result, *Z. wrayi* was categorised as Endangered, while *Z. aurantiacum* and *Z. petiolatum* were categorised as Vulnerable (IUCN 2021). The low abundance of the HCV species might be limited the occurrence of the species in Kelantan. Nevertheless, future conservation status analyses with the Malaysia Red List might provide reliable conservation status of the HCV species in Malaysia.

The present study documented 16 wild ginger species, as shown in Table 3. Wild ginger plants were observed in all study sites, Gua Ikan, Gua Madu, Gunung Reng and Gua Setir. Nonetheless, the gingers in Gunung Reng and Gua Madu were infertile during the survey period. The absence of inflorescence, flowers, and infructescence constrained genus-level identification. Additionally, fewer site observations were conducted in Gunung Reng and Gua Madu. The limited continuous observation was ascribable to the movement control order due to COVID-19 pandemic in Kelantan. Furthermore, the limestone forest in Gua Madu was flooded after prolonged heavy rain as the cave is adjacent to the riverbank of Sungai Galas. The flood affected the plant growth cycle (Striker 2012), which hindered data collection in the study site. Accordingly, continuous site observation in the future is recommended for ginger species identification at Gunung Reng and Gua Madu limestone areas to update the distribution list of limestone gingers in Kelantan.

In the present study, 13 wild ginger species were recorded in Gua Setir and five species in Gua Ikan. The Zingiberaceae species was not documented in a previous floral study at Gua Setir and Gua Ikan in 1990 by Davison and Kiew. Consequently, the current study provided the updated scientific record of Zingiberaceae in Gua Setir and Gua Ikan. The species compositions in Gua Setir and Gua Ikan represented 81.25% and 31.25% of the overall species composition listed in Table 2, respectively. The current study demonstrated that both sites contained different species combinations with 12.5% overlapping species. *Etlingera littoralis* and *E. punicea* were the species distributed in both limestone forests. The remainder, 87.5% ginger species, were localised, found in either limestone forests. A similar floral distribution was recorded in different limestone hills in FELDA Chiku during a floral observation by Kiew *et al*. (2019). The report also indicated that no limestone hill recorded more than 60% of the identified limestone flora.

*Z. ottensii*, a non-native ginger species, was identified at Gua Setir. The domesticated ginger species has been cultivated and utilised as traditional medicine (Theilade 1996). The plant is also employed as ornamentals in Southeast Asia countries (Kizhakkayil & Sasikumar 2011). The present study discovered a single plant of the species approximately 100 m from the limestone karst, which the locals probably introduced.

Environmental factors are vital determinants of floral species richness (Thammanu *et al*. 2021). Wild gingers prefer damp, humid, and shady habitats (Larsen *et al*. 1999). Gua Setir and Gua Ikan demonstrated different environmental conditions. Gua Setir is located in a rural area surrounded by small patches of dense forest. The surrounding cave with streams provided suitable habitat for the gingers. Nonetheless, most nearby areas have been logged and converted into plantations. During the survey, logging activities and abandoned mine lakes were observed near the study site.

Gua Setir cave is accessible by two-wheelers, making the rural cave famous among the locals for guano collection. Davison and Kiew reported similar activities in 1990. Consequently, the anthropogenic activities around the cave are a significant threat to the identified ginger species. Conversely, Gua Ikan is a well-maintained recreational area with a small river crossing over the cave and a forest surrounds a part of the cave. Nevertheless, Gua Ikan exhibited more open areas than Gua Setir. Visitors of the recreational park perform cave exploration, rock climbing, and picnic activities. Frequent human and maintenance activities around the cave limited the occurrence of wild ginger in the study site.

## CONCLUSION

Wild gingers (Zingiberaceae) were surveyed in four limestone forests of Kelantan. Gua Setir and Gua Ikan recorded 16 wild ginger species. The gingers belonging to the species *Z. aurantiacum*, *Z. petiolatum* and *Z. wrayi* were identified as High Conservation Value (HCV) species. The present study has also updated the Kelantan limestone flora with 15 new records of wild gingers. Moreover, differing species composition between the study sites was observed. The finding was supported by Kiew *et al*. (2019) that indicated floral composition variations between limestone hills. Consequently, ginger identification and conservation in each limestone hill are vital for the survival of the wild ginger species. Furthermore, the protection and restoration of limestone ecosystems have been listed as one of the national biodiversity targets in the National Policy of Biodiversity 2016–2025 of Malaysia (Ministry of Natural Resources and Environment 2016). Accordingly, conservation efforts should be implemented along with sustainable management of the limestone forests currently enforced to protect the Zingiberaceae species and comply with the national policy.

## Figures and Tables

**Table 1:** Location of study sites in Kelantan, Peninsular Malaysia.

Location	District	Coordinate	Elevation (m a.s.l)	Observation month
Gua Ikan	Dabong	5°21′12″N; 102°01′32″E	53.29	January 2020–March 2020 September 2020–December 2020 March 2021–April 2021
Gua Madu	Gua Musang	4°50′12″N; 101°56′58″E	103.03	January 2020–March 2020 March 2021
Gua Setir	Jeli	5°39′55″N; 101°55′29″E	86.29	January 2020 June 2020–October 2020 January 2021–May 2021
Gunung Reng	Jeli	5°42′53″N; 101°44′43″E	102.32	February 2020–March 2020 March 2021

*Note:* m a.s.l = meter above sea level.

**Table 2:** Species composition of gingers from limestone forests of Kelantan, Peninsular Malaysia.

No.	Tribes	Species	IUCN Red List	Malaysia Red List
1	Alpinieae	*Alpinia javanica* Blume	LC	NE
2	Alpinieae	*Etlingera littoralis* (J.Koenig)	LC	NE
3	Alpinieae	*Etlingera maingayi* (Baker)	LC	NE
4	Alpinieae	*Etlingera punicea* (Roxb.)	LC	NE
5	Alpinieae	*Plagiostachys* sp.	-	-
6	Alpinieae	*Wurfbainia uliginosa* (J.Koenig) Giseke *(****≡**** Amomum uliginosum* Koenig)	LC	NE
7	Alpinieae	*Sundamomum hastilabium* (Ridl.) A. D. Poulsen & M. F. Newman *(****≡**** Amomum hastilabium* Ridl.)	LC	NE
8	Globbeae	*Globba leucantha* Miq.	NE	NE
9	Globbeae	*Globba patens* Miq.	LC	NE
10	Zingiberaceae	*Bosenbergia plicata* (Ridl. & Ridley) Holttum	NE	NE
11	Zingiberaceae	*Zingiber aurantiacum* (Holttum) Theilade*	VU	NE
12	Zingiberaceae	*Zingiber ottensii* Valeton	DD	NE
13	Zingiberaceae	*Zingiber petiolatum* (Holttum) Theilade*	VU	NE
14	Zingiberaceae	*Zingiber puberulum* Ridl.	NT	NE
15	Zingiberaceae	*Zingiber spectabile* Griff.	DD	NE
16	Zingiberaceae	*Zingiber wrayi* Prain ex Ridl.*	EN	NE

*Notes:* DD = Data Deficient; LC = Least Concern; NT = Near Threatened; VU = Vulnerable; EN = Endangered; NE = Not Evaluated;

* = High Conservation Value (HCV) species.

**Table 3:** Species distribution of gingers at limestone forest of Kelantan, Peninsular Malaysia.

No.	Tribes	Species	Gua Ikan	Gua Setir
1.	Alpinieae	*Alpinia javanica* Blume NR	−	*+*
2.	Alpinieae	*Etlingera littoralis* (J.Koenig) NR	*+*	*+*
3.	Alpinieae	*Etlingera maingayi* (Baker)	*+*	−
4.	Alpinieae	*Etlingera punicea* (Roxb.) NR	*+*	*+*
5.	Alpinieae	*Plagiostachys* sp. NR	−	*+*
6.	Alpinieae	*Wurfbainia uliginosa* (J.Koenig) Giseke NR *(****≡**** Amomum uliginosum* Koenig)	−	*+*
7.	Alpinieae	*Sundamomum hastilabium* (Ridl.) A.D.Poulsen & M.F.Newman NR	*+*	−
8.	Globbeae	*Globba leucantha* Miq. NR	−	*+*
9.	Globbeae	*Globba patens* Miq. NR	−	*+*
10.	Zingiberaceae	*Bosenbergia plicata* (Ridl. & Ridley) Holttum NR	−	*+*
11.	Zingiberaceae	*Zingiber aurantiacum* (Holttum) Theilade NR	−	*+*
12.	Zingiberaceae	*Zingiber ottensii* Valeton*^NR^	−	*+*
13.	Zingiberaceae	*Zingiber petiolatum* (Holttum) Theilade NR	−	*+*
14.	Zingiberaceae	*Zingiber puberulum* Ridl. NR	−	*+*
15.	Zingiberaceae	*Zingiber spectabile* Griff. NR	*+*	−
16.	Zingiberaceae	*Zingiber wrayi* Prain ex Ridl. NR	−	*+*
Total			5	13

*Notes:*

* = not native;

NR = New record to limestone forest of Kelantan state.

## References

[b1-tlsr-33-3-33] Aliaa-Athirah AM, Kiew R, Rafidah AR, Ummul-Nazrah AR, Ong PT (2019). Lessons from the Gua Musang massif for conservation of the Malaysian limestone flora. Conservation Malaysia.

[b2-tlsr-33-3-33] Appalasamy S, Arumugam N, Geng BJ, Rak AE (2019). A short note on wild gingers (Zingiberaceae) in Pulau Pangkor, Perak, Peninsular Malaysia. The Malaysian Forester.

[b3-tlsr-33-3-33] Appalasamy S, Arumugam N (2020). Four new records of Zingiberaceae in Gunung Telapak Burok, Berembun Forest Reserve (Fr), Negeri Sembilan. IOP Conference Series: Earth and Environmental Science.

[b4-tlsr-33-3-33] Appalasamy S, Arumugam N, Lam Y, Nur Azizun R (2020). Wild gingers (Zingiberaceae) at Sungai Kangkawat, Imbak Canyon Conversation Area (ICCA), Sabah. Journal of Tropical Biology and Conversation.

[b5-tlsr-33-3-33] Appalasamy S, Rathamanalan S, Arsogah S, Harni S, Sam KK, Amaludin NA, Geng BJ, Kumaran JV, Arumugam N, Subramaniam S (2020). Gingers species diversity and distribution along a natural trail of Lojing Highlands, Kelantan. IOP Conference Series: Earth and Environmental Science.

[b6-tlsr-33-3-33] Chin SC (1977). The limestone flora of Malaya I. Gardens' Bulletin Singapore.

[b7-tlsr-33-3-33] Chin SC (1983). The limestone flora of Malaya IV. Gardens' Bulletin Singapore.

[b8-tlsr-33-3-33] Davison GWH, Kiew R (1990). Survey of flora and fauna of limestone hills in Kelantan, with recommendations for conservation.

[b9-tlsr-33-3-33] de Boer H, Newman M, Poulsen AD, Droop AJ, Fér T, Hiên LTT, Hlavatá K, Lamxay V, Richardson VE, Steffen K, Leong-Škorničková J (2018). Convergent morphology in Alpinieae (Zingiberaceae): Recircumscribing *Amomum* as a monophyletic genus. Taxon.

[b10-tlsr-33-3-33] HCV Malaysia Toolkit Steering Committee (2018). Malaysia national interpretation for the identification of high conservation values.

[b11-tlsr-33-3-33] Henderson MR (1939). The flora of limestone hills of the Malay Peninsula. Journal of the Malayan Branch of the Royal Asiatic Society.

[b12-tlsr-33-3-33] Holtum RE (1950). The Zingiberaceae of the Malay Peninsula. The Gardens’ Bulletin Singapore.

[b13-tlsr-33-3-33] IUCN (2021). The IUCN red list of threatened species. Version 2021.

[b14-tlsr-33-3-33] Izlamira R, Appalasamy S, Nivaarani A, Noor Hisham MZA, Abdullah MRC, Abong NND, Jemali NJN, Amaludin NA, Nordin SM (2020). Zingiberaceae diversity in Ulu Sat Forest Reserve, Kelantan. Hutan Ulu Sat: Nadi pemeliharaan alam semulajadi Kelantan (Ulu Sat Forest: The heart of Kelantan's nature conservation).

[b15-tlsr-33-3-33] Khaw SH (2001). The genus *Etlingera* (Zingiberaceae) in Peninsular Malaysia including a new species. Gardens' Bulletin Singapore.

[b16-tlsr-33-3-33] Kiew R, Rafidah AR, Ong PT, Ummul-Nazrah AR (2017). Limestone treasures, rare plants in Peninsular Malaysia-what they are, where they grow and how to conserve them. Malaysian Naturalist.

[b17-tlsr-33-3-33] Kiew R, Ummul-Nazrah A, Ong P, Imin K, Aliaa-Athirah A, Rafidah A (2019). Distribution and conservation implications of limestone plant species in Felda Chiku Limestone Flora, Kelantan, Malaysia. Journal of Tropical Forest Science.

[b18-tlsr-33-3-33] Kizhakkayil J, Sasikumar B (2011). Diversity, characterisation and utilisation of ginger: A review. Plant Genetic Resources.

[b19-tlsr-33-3-33] Kress WJ, Prince LM, Williams KJ (2002). The phylogeny and a new classification of the gingers (Zingiberaceae): Evidence from molecular data. American Journal of Botany.

[b20-tlsr-33-3-33] Lamb A, Gobilik J, Ardiyani M, Poulsen AD (2013). A guide to gingers of Borneo.

[b21-tlsr-33-3-33] Larsen K, Ibrahim H, Khaw S, Saw LG (1999). Gingers of Peninsular Malaysia and Singapore.

[b22-tlsr-33-3-33] Liew T-S, Foon J-K, Clements GR (2021a). Conservation of limestone ecosystems of Malaysia, Part I, Acknowledgements, Methodology, Overview of limestone outcrops in Malaysia, References, detailed information on limestone outcrops of the states: Johor, Negeri Sembilan, Terengganu, Selangor, Perlis. Figshare.

[b23-tlsr-33-3-33] Liew T-S, Foon J-K, Clements GR (2021b). Conservation of limestone ecosystems of Malaysia, Part V, Detailed information on limestone outcrops of Kelantan. Figshare.

[b24-tlsr-33-3-33] Liew T-S, Price L, Clements R (2016). Using Google Earth to improve the management of threatened limestone karst ecosystems in Peninsular Malaysia. Tropical Conservation Science.

[b25-tlsr-33-3-33] Ministry of Natural Resources and Environment (NRE) (2016). National policy on biological diversity 2016–2025.

[b26-tlsr-33-3-33] Newman M, Lhuillier A, Poulsen AD (2004). Checklist of the Zingiberaceae of Malesia. Blumea.

[b27-tlsr-33-3-33] Poulsen AD (2006). Etlingera of Borneo.

[b28-tlsr-33-3-33] Sam YY, Ibrahim H (2016). A new *Globba* with large white floral bracts from Peninsular Malaysia. PhytoKeys.

[b29-tlsr-33-3-33] Sam YY, Saw LG (2005). Three new species of *Scaphochlamys* (Zingiberaceae) from Peninsular Malaysia. Gardens’ Bulletin Singapore.

[b30-tlsr-33-3-33] Sam YY, Ibrahim H, Saw LG (2015). Four new species of *Scaphochlamys* (Zingiberaceae) from Peninsular Malaysia. Phytotaxa.

[b31-tlsr-33-3-33] Smith RM (1990). Alpinia (Zingiberaceae): A proposed new infrageneric classification. Edinburgh Journal of Botany.

[b32-tlsr-33-3-33] Striker GG, Mworia JK (2012). Flooding stress on plants: Anatomical, morphological and physiological responses. Botany.

[b33-tlsr-33-3-33] Thammanu S, Marod D, Han H, Bhusal N, Asanok L, Ketdee P, Gaewsingha N, Lee S, Chung J (2021). The influence of environmental factors on species composition and distribution in a community forest in Northern Thailand. Journal of Forestry Research.

[b34-tlsr-33-3-33] Theilade I (1996). Revision of the genus *Zingiber* in Peninsular Malaysia. The Gardens' Bulletin Singapore.

[b35-tlsr-33-3-33] Theilade I (1999). A synopsis of the genus *Zingiber* (Zingiberaceae) in Thailand. Nordic Journal of Botany.

[b36-tlsr-33-3-33] Xu Z, Chang L (2017). Zingiberaceae. Identification and control of common weeds (3).

[b37-tlsr-33-3-33] Yong WSY, Chua LSL, Lau KH, Siti-Nur Fatinah K, Cheah YH, Yao TL, Rafidah AR, Lim CL, Syahida-Emiza S, Ummul-Nazrah AR (2021). Malaysia red list: Plants of Peninsular Malaysia (IPart I).

